# The Characterization of Optical Fibers for Distributed Cryogenic Temperature Monitoring

**DOI:** 10.3390/s22114009

**Published:** 2022-05-25

**Authors:** Leonardo Marcon, Antonella Chiuchiolo, Bernardo Castaldo, Hugues Bajas, Andrea Galtarossa, Marta Bajko, Luca Palmieri

**Affiliations:** 1 Department of Information Engineering, University of Padova, Via G.Gradenigo 6/B, 35131 Padova, Italy; andrea.galtarossa@unipd.it; 2 CERN—European Organization for Nuclear Research, Espl. des Particules 1, 1211 Meyrin, Switzerland; castaldobernardo@gmail.com (B.C.); hugues.bajas@psi.ch (H.B.); marta.bajko@cern.ch (M.B.); 3 GSI Helmholtzzentrum für Schwerionenforschung GmbH, Planckstraße 1, 64291 Darmstadt, Germany; antonella.chiuchiolo@cern.ch

**Keywords:** optical fibers, Rayleigh scattering, distributed sensing, cryogenic temperature, coating, polymers, superconducting links

## Abstract

Thanks to their characteristics, optical fiber sensors are an ideal solution for sensing applications at cryogenic temperatures, such as the monitoring of superconducting devices. Their applicability at such temperatures, however, is not immediate as optical fibers exhibit a non-linear thermal response which becomes rapidly negligible below 50 K. A thorough analysis of such a response down to cryogenic temperatures then becomes necessary to correctly translate the optical interrogation readings into the actual fiber temperature. Moreover, to increase the fiber sensitivity down to a few kelvin, special coatings can be used. In this manuscript we described the thermal responses experimental characterization of four commercially available optical fiber samples with different polymeric coatings in the temperature range from 5 K to 300 K: two with acrylate coatings of different thickness, one with a polyimide coating and one with a polyether–ether–ketone (PEEK) coating. Multiple thermal cycles were performed consecutively to guarantee the quality of the results and a proper estimate of the sensitivity of the various samples. Finally, we experimentally validated the quality of the measured thermal responses by monitoring the cool down of a dummy superconducting link from room temperature to approximately 50 K using two fibers coated, respectively, in acrylate and PEEK. The temperatures measured with the fibers agreed and matched those obtained by standard electronic sensors, providing, at the same time, further insight in to the cool-down evolution along the cryostat.

## 1. Introduction

The increasing interest toward optical fiber sensors, in both research and commercial fields, is driven by their excellent properties, such as reduced dimensions, electromagnetic-interference immunity, resistance to harsh environments and, eventually, the possibility of performing distributed measurements. All these properties make optical fiber sensors ideal candidates for the monitoring of superconducting devices as they can be integrated into the structure and they can withstand the cryogenic temperatures, high magnetic fields, gas flows and radioactivity levels that are typical of such environments [1]. Successful early demonstrations of cryogenic monitoring by means of optical fiber sensors have been performed using both single point optical fiber sensors, namely fiber Bragg gratings (FBG) [2] and distributed optical fiber sensors (DOFS) based on both Rayleigh [3] and Brillouin [4] scattering. In particular, setups implementing optical frequency-domain reflectometry (OFDR) based on Rayleigh backscattering, have been recently attracting increasing attention [3,5] due to the possibility of performing dynamic measurements with a high acoustic bandwidth and spatial resolution [6].

The implementation of temperature or strain monitoring systems for superconducting devices, based on either FBGs or DOFS, is not straightforward because the thermal response of standard fibers exhibits a non-linear behavior for decreasing temperatures and becomes rapidly negligible below 50 K [2,7]. In fact, several phenomena contribute to the fiber thermal response—mostly the thermo-optic effect, the thermo-elastic effect and the strain between the fiber and the coating—and their contribution to the thermal response change as a function of the temperature. An accurate thermal response characterization covering the temperature range from room temperatures to cryogenic ones is then necessary to correctly map the results of the optical interrogation to the actual fiber temperature. Different solutions have been identified over the years, addressing both the non-linear response and the reduced sensitivity at low temperatures. For FBG systems, a calibration over a whole thermal cycle performed in a controlled environment allowed the evaluation of the non-linear response and mapped the FBG wavelength shift to the corresponding absolute temperature [8]. Moreover, the use of different substrates, or of special coatings made with different polymers and/or with different thicknesses, have been proven effective to increase the fiber sensitivity even at temperatures as low as a few kelvin [8,9]. Similar solutions have also been explored for DOFS systems, using standard optical fibers [2], and interesting results have been achieved in the monitoring of dynamic vibrations and quenches in high temperature superconductive magnets [5,10,11]. These early results highlighted that the actual sensitivity of the optical fibers depended on the material and thickness of the polymeric coating applied to the fiber.

In this manuscript we expand the preliminary analysis initiated in [2,12] by analyzing in more detail the experimental thermal response characterization of four commercially available optical fiber samples with different polymeric coatings, i.e., acrylate of thickness 62.5 μm and 137.5 μm, polyimide of thickness 15 μm and polyether–ether–ketone (PEEK) of thickness 137.5 μm; in all cases the diameter of the silica fiber was 125 μm. The temperature range used to perform the characterization ranged from 5 K to 300 K. The fiber samples were monitored during multiple consecutive thermal cycles to guarantee the consistency of the results and a proper estimate of the different sample sensitivities. Results showed that the overall sensitivity was mainly related to the coating thickness, with thicker coatings yielding larger sensitivity. On the contrary, the choice of the material only had a marginal impact on the linearity of the response. We experimentally validated the quality of the measured thermal responses by using optical fibers coated with the same materials to monitor the temperature of a dummy superconducting link during the cool down from room temperature to approximately 50 K. Two fibers were deployed together with standard electrical sensors used as a reference and their results were compared. An excellent agreement between the temperatures measured by optical and electrical sensors was observed over the whole temperature range. Moreover, the optical sensors provided further insights to the temperature evolution inside the cryostat during the cool down, thanks to their ability to monitor the link along its whole length.

## 2. Overview of the Technique and of the Theoretical Model

In optical frequency domain reflectometry (OFDR) the probe signal is generated by linearly sweeping the central wavelength of a highly coherent laser source over a wide optical band of several tens of nanometers. The probe signal is partially coupled to the fiber under test (FUT), and partially used as a local oscillator (LO). The LO is mixed, before detection, with the Rayleigh backscattering generated by the probe signal in the FUT. This heterodyne detection and the wide optical band swept by the laser source, guarantee the very high sensitivity and millimetric spatial sampling, which are characteristic of the OFDR technique. As long as the fiber is not heated above the critical temperature (typically well above 800 ${}^{{}^{\circ}}C$ [13]), consecutive interrogations of the FUT at rest always result in the same output trace, which can then be considered as the fiber’s own fingerprint. However, if a perturbation such as temperature or strain variation acts on the fiber, the spectrum of the fiber fingerprint undergoes a frequency shift proportional to the intensity of the perturbation; therefore, by measuring the shift, we can monitor the perturbation. OFDR-based distributed sensing is thus performed by acquiring a reference trace when the fiber is at rest, and then identifying the local shifts of the fingerprint spectrum comparing the reference trace with consecutive traces acquired when stresses are acting on the fiber. It is important to highlight, however, that since temperature changes and strains cause cumulative shifts, if both perturbations act simultaneously in the same position of the fiber, the identification of the single perturbations becomes harder, and further information is necessary to completely discriminate them [14]. The analysis between the reference and stressed traces is performed by comparing corresponding sections of both traces. The length of this section takes the name of *sensor gauge*, and physically determines the spatial resolution of the measurement. The section then moves along the traces according to a different parameter called *sensor spacing*.

We start by taking into consideration an optical fiber with a generic polymeric coating at an initial temperature $T_{0}$, deployed unbounded from any structure and free of strain. As introduced before, if the temperature changes to *T*, the Rayleigh spectrum exhibits a wavelength shift

(1) $$\delta \lambda =\Lambda (T,T_{0})$$

where $\Lambda (T,T_{0})$ is the response function of the fiber at temperature *T* with respect to the initial temperature $T_{0}$. There are three different effects that contribute to $\Lambda (T,T_{0})$: (i) the silica thermo-optic effect, which locally changes the fiber’s optical properties as a function of the silica temperature; (ii) the silica thermo-elastic effect that deals with all the stresses generated by silica’s thermal contractions; (iii) the strain induced on the silica component of the fiber by the greater thermal contraction of the polymeric coating. For temperatures T>250 K, or around room temperature in general, the function $\Lambda (T,T_{0})$ exhibits a linear behaviour, however, for progressively lower temperatures *T*, the thermo-optic component and the thermo-elastic component start exhibiting non-linear behaviours, till they become almost negligible for T≤50 K [7]. Common polymeric coatings continue to contract until lower temperatures are reached, so as long as the coating keeps its grip on the silica surface, its contraction drags the underlying molecules, inducing a measurable strain. When approaching absolute zero, the coating will eventually stop contracting, and the function $\Lambda (T,T_{0})$ approaches an horizontal asymptote. The choice of the coating polymer based on its mechanical and thermal properties then becomes fundamental when using the fibers as a distributed sensor to monitor cryogenic environments, as it directly influences the sensitivity:

(2) $$S=\frac{\partial \Lambda (T,T_{0})}{\partial T}.$$

To actually map the thermal response of the fiber to the absolute temperature of the environment surrounding the fiber it is then necessary to compute the inverse of $\Lambda (T,T_{0})$:

(3) $$T=\Lambda^{-1}\left(\delta \lambda ,T_{0}\right)\;.$$

The function $\Lambda^{-1}\left(\delta \lambda ,T_{0}\right)$ is characteristic of the specific combination of the fiber with that particular coating and of the particular initial temperature $T_{0}$. If after the calibration the measurement is performed with respect to a different initial reference temperature $T_{1}$, the measured wavelength shift should be corrected by the quantity $\Lambda (T_{1},T_{0})$, so that the actual temperature is given by

(4) $$T=\Lambda^{-1}\left(\delta \lambda +\Lambda \left(T_{1},T_{0}\right),T_{0}\right)\;.$$

## 3. Experimental Characterization of the Fibers’ Thermal Responses

This section describes the first experiment, during which the thermal responses and relative sensitivities of four fiber samples with different coatings have been characterized. Using a closed-cycle refrigerator system, the characterization was performed in the range from 15 K to 300 K. The results showed that the sensitivity was largely determined by the coating thickness and only marginally by the material.

### 3.1. Experimental Setup

The experimental characterization of the curves $\Lambda (T,T_{0})$, for $T_{0}=300\;K$, have been performed for four optical fiber samples of length L=10m, with different polymer coatings of different thermal properties and thicknesses, to evaluate both the thermal responses at cryogenic temperatures and the influence of the coating on the performance of the sensor. The diameter of the silica fibers in all cases was 125 μm. Two of the four fiber samples considered in this experiment were standard telecommunication fiber with a bi-layer acrylate coating with different thickness of 62.5 μm (Pirelli, FreeLight) and 137.5 μm (Taihan Fiberoptics, custom made for the experiment); another sample was a common sensor-oriented fiber with a very thin polyimide coating of thickness 15 μm (OFS, GEOSIL-SM); finally, the last sample was a special fiber coated with an inner layer of polyimide with thickness 16 μm, overcoated with a 121.5 μm thick layer of polyether–ether–ketone, also known as PEEK (Zeus, PEEK Fiber). Hereinafter these samples are addressed by the labels listed in Table 1.

**Figure 1:**
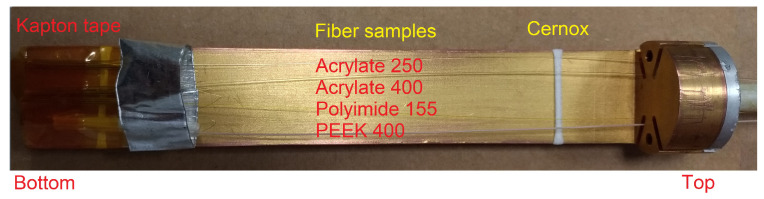
Arrangement of the fiber samples over the cryocooler platform. The fibers enter the platform from the holder on the top and were fixed at the bottom with caption tape. The fiber samples were, from the top as follows: Acrylate250, Acrylate400, Polyimide155, PEEK400.

The fiber samples were deployed inside the “cryo-cooler”, a closed-cycle refrigerator system which included a pulse tube and a cryogen-free sealed variable temperature insert (VTI) of 50 mm inner diameter and 200 mm height [15], in which the samples were cooled by cold helium gas after pumping the void VTI volume. The fiber samples were loosely suspended over a gold-coated copper platform of dimensions 24 mm width and 160 mm length, and fixed at the end by mean of kapton tape, as shown in Figure 1. In the same figure it is possible to see the white tape holding the standard reference electrical resistive sensors (CERNOX) that was placed after 20 mm from the beginning of the platform. The ring made of aluminum tape, visible close to the kapton tape at the bottom part of the platform, was used for further protection during the installation.

**Figure 2:**
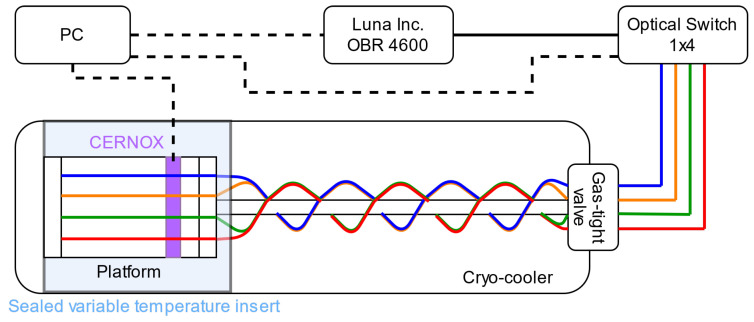
Schematic representation of the setup. Dashed lines represent electrical connections, the colored lines represent different fiber samples, namely blue, red, green and orange for Acrylate250, Acrylate400, Polyimide155 and PEEK400, respectively. The fibers in the platform were monitored through an optical switch connected to the Luna Inc. OBR 4600. A PC synchronized the OBR, the optical switch and the readings of the CERNOX inside the variable temperature insert.

The optical setup is schematically represented in Figure 2 and shows that the four fiber samples were measured using a 4×1 optical switch preceeding the OBR 4600, produced by Luna Inc. (Roanoke, VA, USA). The optical connection between the fiber samples and the outside of the cryostat was achieved thanks to a dedicated gas-tight valve. The OBR and the switch were controlled through a dedicated custom script that consecutively measured all four fiber samples every two minutes. The OBR was setup to achieve the best spatial sampling possible of approximately 10 μm, by setting the scanned bandwith to 88 nm at a scan rate of 200 nm/s, thus requiring 0.44 s to scan each fiber sample. This time interval was short enough to consider the temperature of the fiber as constant through the whole measurement. The Rayleigh spectra collected during the acquisitions were analyzed with a spacing of 2.5 mm and using a sensor gauge of 5 mm, which corresponded to the actual spatial resolution. Given the very dense spatial sampling, such a gauge length was long enough to ensure the quality of the wavelength shift estimation, but at the same time was short enough to avoid measurement artifacts due to the small temperature gradient possibly present between the tip and the end of the platform. To guarantee the highest accuracy, the wavelength shifts δλ used for the characterization of the thermal response $\Lambda (T,T_{0})$ were measured along a section with a length of 2 cm, centered in correspondence with the CERNOX sensor.

**Figure 3:**
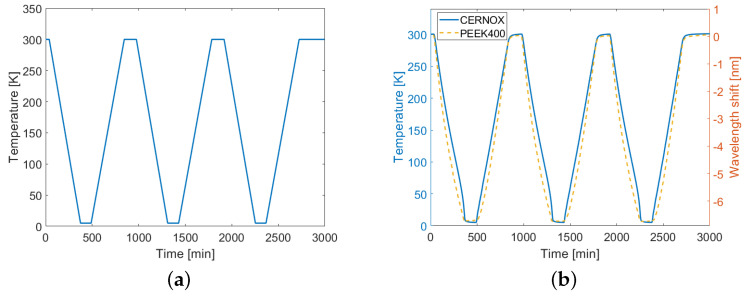
(**a**) Nominal thermal cycles of the cryo-cooler. (**b**) Comparison between the absolute temperatures measured with the CERNOX (left axis) and the wavelength shifts measured on the PEEK400 sample with respect to the reference temperature $T_{0}=300\;K$ (right axis).

The cryo-cooler was set up to perform three full thermal cycles between room temperature, $T_{0}=300\;K$, and $T_{min}=5\;K$—the lowest temperature achievable by the cryo-cooler—following the nominal pattern shown in Figure 3a. All full thermal cycles consisted of a cool down and a warm up, each composed of a six-hour-long temperature ramp followed by a two-hour plateau at a constant temperature. Such long cycles were necessary to avoid ripples caused by thermal inertia, especially close to $T_{0}$ and $T_{min}$ that would have reduced the quality of the measurement results. The aim of monitoring the fiber samples over different thermal cycles was dual. It was firstly used to verify that even when exposed to such low temperatures no peeling occurred, and, secondly, to collect multiple replicas of the fiber’s behavior to further increase the reliability and accuracy of the measured data. The peeling, i.e., the loss of grip between the coating and the fiber below, would have caused hysteresis between the data collected over different thermal cycles and a significant decrease in the sensitivity of the corresponding fiber sample.

### 3.2. Results

After the temperature inside the VTI was stabilized at $T_{0}=300\;K$, the reference traces for all four fiber samples were collected, and the measurement routine started. The variation over time of the actual VTI temperature was measured by the reference CERNOX, as reported in Figure 3b, with respect to the left vertical axis. The temperature followed the nominal thermal cycles with the exception of some deviations, especially near the plateaus. Actually, when approaching the highest temperatures, the automatic control slowed down the warming to avoid ripples, whereas when approaching the lowest temperatures (below 50 K) the decreased thermal capacity of the elements inside the VTI caused faster temperature changes. As an example, Figure 3b also shows the wavelength shift measured on the PEEK400 sample at the same position of the CERNOX; data were referred to the right vertical axis. While the correlation of the wavelength shift with respect to the VTI temperature was evident, it could also be seen that the two curves did not completely overlap because of the non-linear behaviour of the fiber thermal response.

**Figure 4:**
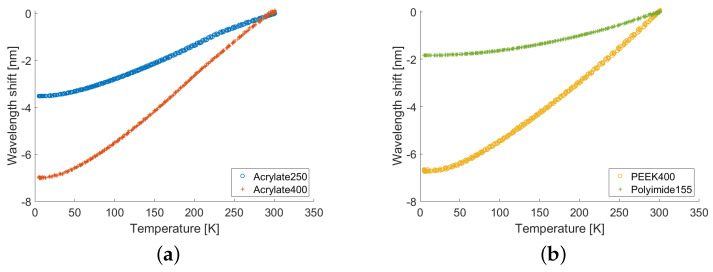
(**a**) Experimentally measured $\Lambda (T,T_{0}=300\;K)$ for the Acrylate250 and Acrylate400 samples over three thermal cycles. (**b**) Experimentally measured $\Lambda (T,T_{0}=300\;K)$ for the PEEK400 and Polyimide155 samples over three thermal cycles.

Figure 4 shows, for each fiber sample, the wavelength shifts measured during the full thermal cycle as a function of the corresponding VTI temperature measured by the CERNOX. In more detail, we considered a section of approximately 2 cm in length of fiber, centered in correspondence with CERNOX; since the sensor spacing was set to 2.5 mm, this yielded eight measurements of the wavelength shift, which were averaged to reduce the noise; these averages are the values reported in Figure 4. These curves basically represent the superposition of six experimental measures of the thermal responses $\Lambda \left(T,T_{0}=300K\right)$, three of which were acquired during the cool downs and the other three during the warm ups. The fact that the curves overlapp almost perfectly proved the absence of any significant hysteresis, confirming that the coatings kept their grip on the silica during the whole measurement. More quantitatively, the maximum differences between the wavelength shifts measured at the same temperature for the full thermal cycle are reported in Table 2, along with the relative weight of these differences with respect to the measured maximum wavelength shifts; as can be seen, this difference was at most 2%. We also remark that these results were consistent with previously reported measurements obtained in similar conditions [2,3].

**Figure 5:**
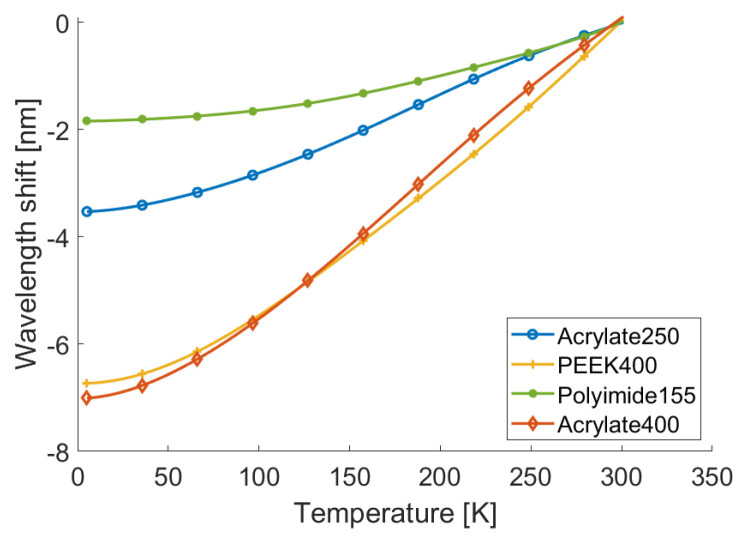
Thermal response functions $\Lambda (T,T_{0}=300\;K)$ of all the fiber samples.

To estimate the thermal response functions $\Lambda (T,T_{0})$, the experimental curves shown in Figure 4 were fitted with polynomials of sixth degree, with the constraint that their derivative was positive. This constraint was set to avoid the fitting producing non-physical non-monotonic trends, especially at cryogenic temperatures where the noise was higher. The best fitting polynomials are plotted in Figure 5 and show a strong agreement with the curves in Figure 4; actually, the root-mean square interpolation residuals were less than approximately 0.5% of the measured wavelength-shift range, as reported in Table 2. As can be seen in Figure 5, a strong correlation between the coating thickness and the magnitude of the thermal response of all fiber samples was evident. In particular, focusing, for instance, on the Acrylate400 and PEEK400 samples, it was interesting to observe how different coating materials with the same thickness exhibited responses with very similar magnitudes and marginally different shapes. A qualitative explanation of this behavior could be given by noting that the mechanical interaction between fiber and coating was controlled by the relative stiffness, which was proportional to the young modulus multiplied by the cross section. Increasing the thickness of the coating increased the stiffness and, consequently, it increased the stress that the thermo-elastic effect of the coating exerted on the fiber. The details of this mechanism depended on the specific property and structure of the coating and, thus, justified the small differences among the samples.

**Figure 6:**
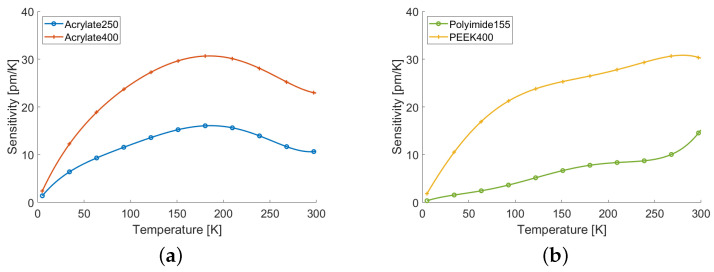
(**a**) Sensitivities of the acrylate-coated fibers. (**b**) Sensitivity of the peek- and polyimide-coated fibers.

As defined in Equation (2), the sensitivity *S* was the first order derivative of the thermal response function with respect to temperature. Figure 6 shows the sensitivities evaluated as formal derivatives of the fitting polynomials. It was interesting to note that the sensitivity of the sample of Acrylate400 was basically a scaled-up version of that of the Acrylate250, and had a maximum at approximately 190 K; on the contrary, the sensitivity of the other two samples was almost monotonic. As expected, in all cases the sensitivities dropped to zero as the temperature decreased at a rate strongly correlated with the coating thickness. In general, however, it was clear that thicker coatings guaranteed higher sensitivity along the whole temperature range. All these observations suggested that it was the material itself that mostly defined the shape of the thermal response, whereas the thickness influenced the magnitude and, thus, the actual sensitivity. All fiber samples with the exception of Polyimide155, exhibited sensitivities higher than 5 pm/K for temperatures higher than 25 K. The samples of Acrylate400 and PEEK400 managed to keep such good sensitivity (respectively, 5 pm/K and 6 pm/K) even down to temperatures of 15 K, which is quite useful range for several cryogenic applications.

The sensitivity *S* quantified the ability of the sensor to detect a temperature variation. Another important parameter was the uncertainty with which the absolute value of the temperature was measured. To a first order approximation, the uncertainty can be expressed as

**Figure 7:**
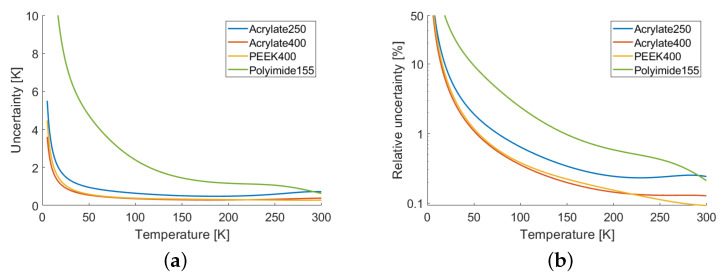
(**a**) Absolute uncertainties and (**b**) relative uncertainties, in log scale.

(5) U=δr/|S|

where δr is the standard deviation of the wavelength shift $\Lambda (T,T_{0})$ measurement. This quantity was affected not only by the accuracy of the instrument measuring the spectral shift, but also on the adequacy of the fitting model. In fact, a good estimate of δr is given by the RMS of the residuals of interpolation, that is the standard deviation of the differences between the experimental data and the fitting curves. These values are reported in Table 1 and, along with the curves of Figure 6, they allowed the calculation of the uncertainties shown in Figure 7. As can be seen, the thicker samples, Acrylate400 and PEEK400, had less than 1% of relative uncertainty for temperatures above 55 K and less than 10% for temperatures above 16 K. The performances of the Acrylate250 sample were somewhat worse, nevertheless, it was noted that this was largely available standard telecommunication fiber. Finally, it was confirmed that the Polyimide250 sample was less adequate for cryogenic applications.

## 4. Superconducting Link

In the second experiment described in this section, two fibers of the kind characterized before were deployed along a cryostat for a superconducting link, and were used to perform distributed temperature monitoring during the cool down. The high spatial resolution of the technique enabled the measurement of marked local temperature variations. These were clearly visible when the link had not reached the thermal equilibrium and were most likely due to non-uniform thermal inertia along the cryostat.

### 4.1. Experimental Setup

With the high-luminosity upgrade of the large hadron collider (LHC) machine approaching [16,17], innovative solutions based on superconducting (SC) links to supply high powerd from remote distances to the LHC magnets were developed [18]. These solutions foresaw the need to build power converters hundreds of meters away from the actual LHC tunnel, and then to supply the current to the magnets via SC links containing multiple superconducting cables connected to their respective circuits. In this experiment the cool down of a dummy SC link, built for testing purposes, was monitored by means of both electrical and optical temperature sensors.

The dummy SC link consisted of a 60-meter-long cylindrical cryostat connected to a helium-based, close-circuit, cryogenic system. After the vacuum pumping inside the cryostat, supercritical helium was flown at a constant rate from the feed box at one end toward the return box at the other. Hereinafter, we referred to a longitudinal coordinate *z* whose zero is at the return box; accordingly, the helium flowed toward the lower values of *z*. Inside the cryostat a dummy cable made of a cheaper materials, closely matching the thermal properties of the superconducting cable, was inserted and fixed in such a way that it did not touch the cryostat walls. Starting at z=20 m, and spaced every 5 m one from the other, five electrical temperature sensors (CERNOX) were placed close to the dummy cable to monitor its temperature evolution during the cool down. The cool down was monitored simultaneously by two different optical fibers, one was a sample of the Acrylate250 described above, while the other was a sample of PEEK400 (see Table 1). The Acrylate250 fiber was 55 m long—limited by the measurement range of the interrogator, considering also the external optical circuit—while the PEEK400 sample was 24 m long, due to limited availability. The fibers were deployed over a flat, uniform, semi-rigid plastic ribbon and kept in position by little plastic rings, equally spaced at approximately 1 m from each other. The rings were loose and a few millimeter wide to keep the fibers in position without applying any strain to them. The fibers were connected to the outside by means of a gas-tight valve with integrated APC optical connectors. During the deployment of the system the Acrylate250 fiber accidentally broke at z=28.5 m and consequently spliced; an all-plastic sleeve was used to protect the splice.

**Figure 8:**
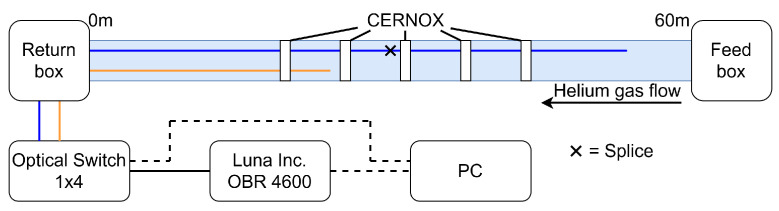
Schematic representation of the SC-Link setup. Dashed lines represent electrical connections, the colored lines represent different fiber samples: blue = Acrylate250 (55 m), orange = PEEK400 (24 m). The fibers were placed inside the SC-Link cryostat starting from the return box. The fibers were monitored by a Luna Inc. OBR4600 through an optical switch. A PC synchronized the OBR and the switch.

The fibers were interrogated by means of a Luna Inc. OBR 4600 instrument that guaranteed a measurement range of 70 m, with a sampling resolution of about 20 μm. The sweep rate was set to 200 nm/s and the scan bandwidth to 88 nm, thus defining a measurement time of about 0.44 s, which was short enough to consider the temperature profile along the fibers constant during the measurement. The OBR was connected to the common port of a 1×4 optical switch to which the two fibers coming out of the cryostat were connected. A computer was connected to both the OBR and the switch to properly synchronize the devices, allowing the consecutive measurement of both fibers. The fibers were monitored along their whole length with a sensor gauge of 10 cm and a spacing of 3 cm. to guarantee the highest measurement quality possible. Given the slow evolution of the cool down, a sampling period of one minute was chosen, but with sufficient computational power, real time monitoring of the temperature would have been a possible alternative. A schematic representation of the experimental setup is shown in Figure 8.

The SC link was cooled from an initial environmental temperature of 297 K down to almost the operational temperature of 50 K. The wavelength shifts measured by the OBR were converted into temperatures using the thermal response functions measured in the previous experiment and shown in Figure 5. Moreover, these responses were corrected, as described by Equation (4), to compensate for the difference of 3 K between the reference temperature of the calibration and that of these measurements.

### 4.2. Results

**Figure 9:**
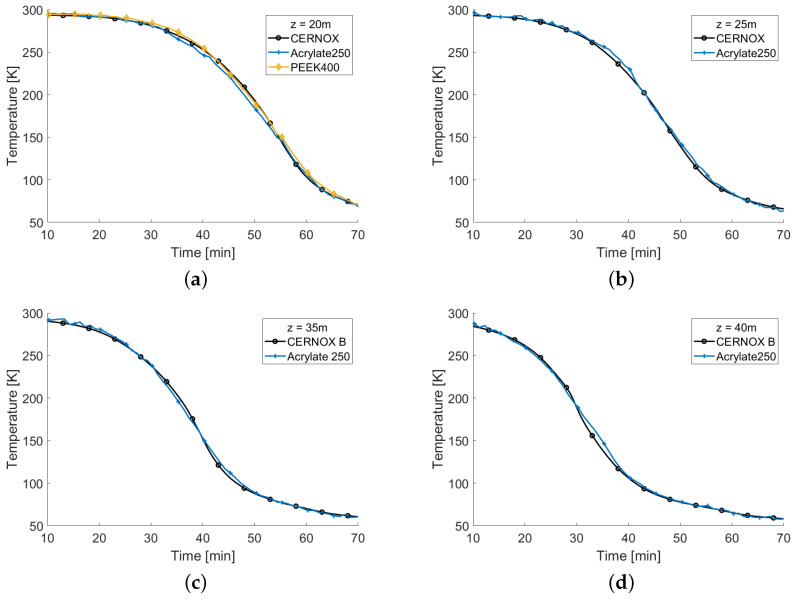
Comparison between temperatures measured with the fibers and that measured by the CERNOX at z=20 m (**a**), 25 m (**b**), 35 m (**c**), and 40 m (**d**).

Due to a delay, the measurements of the fibers started 10 min after the beginning of the cool down; moreover, the cool down had to be interrupted before reaching the target temperature because of an unexpected leakage in the helium circuit. Figure 9 shows the temperatures measured as a function of time at the positions of four of the five CERNOX (the reason why one position was not reported is discussed below). In all the graphs, the black curve refers to the corresponding CERNOX, the blue curve refers to the measurement performed with the Acrylate250 fiber and the yellow one to the PEEK400. Owing to the shorter length of this last sample, PEEK400 was present only in Figure 9a, which refers to the only position shared with the CERNOX. As can be seen, the temperatures monitored with both fibers were in very good agreement with the CERNOX. The non-perfect overlapping of the curves could be partly attributed to the different thermal capacities of the two sensors. While the fiber was very light and hence sensitive to temperature changes, the CERNOXs had a higher thermal inertia that allowed for a smoother temperature reading. From the figures it can also be observed that the SC link cooled down fairly quickly at the beginning, only to slow down when the temperature dropped below 100 K. Even at these temperature, however, the fibers and the CERNOX reading had a remarkable agreement.

**Figure 10:**
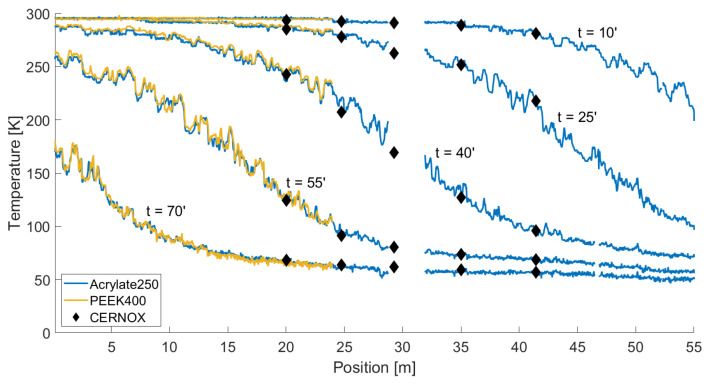
Temperature of the fibers along the whole SC link at different time instants.

**Figure 11:**
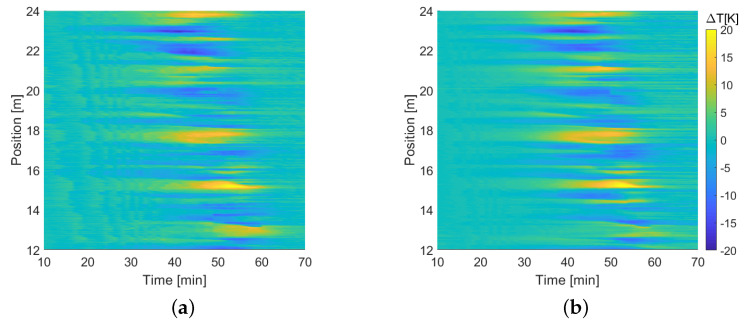
Temperature fluctuations as a function of time and position, along a subsection of the SC link, as measured by (**a**) the Acrylate250 sample, and (**b**) the PEEK400 one.

The main advantage of using distributed sensors based on optical fibers instead of point ones is clearly visible in Figure 10, where the temperatures measured by the two fibers were plotted as a function of distance along the link for different time instants; in the same figure, the black diamonds indicate the position and temperatures read by the CERNOX. Several features of the figure deserve discussion. To begin with, the data for the sample of Acrylate250 have an interruption of a few meters around a distance of 30 m—the shorter PEEK400 sample ended before that position. This was right after the splice mentioned above that had to be performed on the fiber. For a few meters after that splice the interrogator was not able to track the frequency shift. We believe that this was due to some mechanical coupling that was accidentally induced during the installation between the fiber and the cryostat structure, caused by the jutting splice sleeve. Due to this the fiber was subjected to exceedingly large strain that could not be tracked by the OBR. Nonetheless, the optical continuity was not compromised and hence the measurement was still possible beyond that critical section. The second striking feature was that the temperature along the cryostat was clearly not smoothly decreasing from the return box toward the feed box as expected, but exhibited rough fluctuations along the position, with peaks and valleys as high as 20 K. These fluctuations were highlighted for the two fibers separately and for a subsection of the link in Figure 11, where the difference between the actual temperature and the temperature obtained after applying a sliding average over 5 m was reported as a function of time and distance. Despite their random aspect, it was remarkable that these fluctuations were almost the same for both fibers. A similar feature was observed in a similar measurement reported in Ref. [2], performed on a shorter yet similar SC link. In that case, only one fiber was used and the fluctuations were attributed to mechanical issues related to the coating. Nevertheless, the fact that here the same fluctuations affected two completely different fibers ruled out that hypothesis. We could also exclude that the fluctuations were due to vibrations of the system; since the measurement lasted about 0.44 s, any vibration would have totally compromised the measurement, yielding completely unreliable values. Our explanation is that these fluctuations were most likely caused by the non-uniform thermal mass inside the cryostat and by the positioning of the fiber. Due to the sensitivity of the fiber and its little thermal mass, in some points of the cryostat more material was accumulated during the preparation of the setup, or something was shielding the fibers from the helium flow, the temperature at that particular position could have significant local variation. A further support to this hypothesis is the fact that as the cryostat reached the new thermal equilibrium, the fluctuations tended to decrease in amplitude, eventually disappearing as clearly visible at the end of the cool down ($t=70{}^{\prime }$) in the coldest part of the link.

## 5. Conclusions

In this manuscript the experimental thermal responses and sensitivities of four optical fiber samples with different polymeric coatings were characterized at cryogenic temperatures. The results confirmed and extended to other materials the results reported in [2]. Moreover, in observing the thermal responses of the different samples, a correlation between the thickness of the coating and the sensitivities of the fiber was highlighted. Even if the results confirmed a general sensitivity loss at very low temperatures, they allowed us to quantify the advantages offered by thicker coatings. Further work will be needed to better understand how the properties of the coating, such as its optimal thickness or structure, influence the sensor performance. After this calibration procedure, in a second experiment described in this work, we monitored, with two different optical fibers, the cool down of a dummy superconducting link, from room temperature to a final temperature of about 50 K. The results highlighted unexpected temperature fluctuations along the link, on a scale of tens of centimeters and with amplitudes as high as 20 K. We believe that these fluctuations were a consequence of the uneven cooling of the structure due to the uneven distribution of the masses; indeed, the phenomenon was visible only during the transient phase and eventually disappeared once the thermal equilibrium was reached. The results of the reported experiments confirmed that Rayleigh-based distributed temperature sensing was a viable solution for the accurate and detailed monitoring of complex structures at cryogenic temperatures.

## Figures and Tables

**Table 1:** Fiber samples.

Label	Coating Polymer	Coating Thickness [μm]	Fiber Total Diameter [μm]
Acrylate250	Acrylate	62.5	250
Acrylate400	Acrylate	137.5	400
Polyimide155	Polyimide	15.0	155
PEEK400	Polyether–ether–ketone (PEEK)	137.5	400

**Table 2:** Maximum wavelength-shift differences and interpolation residuals, absolute and relative to the measured wavelength-shift range.

	Acrylate250	Acrylate400	PEEK400	Polyimide155
Max. difference among cycles	0.051 nm	0.072 nm	0.077 nm	0.037 nm
	1.46%	1.02%	1.14%	2.03%
Interpolation residual (RMS)	0.0077 nm	0.0084 nm	0.0096 nm	0.0087 nm
	0.22%	0.12%	0.52%	0.12%

## Data Availability

Not applicable.
